# Predicting drug-disease interactions by semi-supervised graph cut algorithm and three-layer data integration

**DOI:** 10.1186/s12920-017-0311-0

**Published:** 2017-12-28

**Authors:** Guangsheng Wu, Juan Liu, Caihua Wang

**Affiliations:** State Key Laboratory of Software Engineering, School of Computer Science, Wuhan University, Wuhan, 430072 People’s Republic of China

**Keywords:** Drug-disease interaction, Integration strategy, Similarity, Graph cut, Guilt-by-association

## Abstract

**Background:**

Prediction of drug-disease interactions is promising for either drug repositioning or disease treatment fields. The discovery of novel drug-disease interactions, on one hand can help to find novel indictions for the approved drugs; on the other hand can provide new therapeutic approaches for the diseases. Recently, computational methods for finding drug-disease interactions have attracted lots of attention because of their far more higher efficiency and lower cost than the traditional wet experiment methods. However, they still face several challenges, such as the organization of the heterogeneous data, the performance of the model, and so on.

**Methods:**

In this work, we present to hierarchically integrate the heterogeneous data into three layers. The drug-drug and disease-disease similarities are first calculated separately in each layer, and then the similarities from three layers are linearly fused into comprehensive drug similarities and disease similarities, which can then be used to measure the similarities between two drug-disease pairs. We construct a novel weighted drug-disease pair network, where a node is a drug-disease pair with known or unknown treatment relation, an edge represents the node-node relation which is weighted with the similarity score between two pairs. Now that similar drug-disease pairs are supposed to show similar treatment patterns, we can find the optimal graph cut of the network. The drug-disease pair with unknown relation can then be considered to have similar treatment relation with that within the same cut. Therefore, we develop a semi-supervised graph cut algorithm, SSGC, to find the optimal graph cut, based on which we can identify the potential drug-disease treatment interactions.

**Results:**

By comparing with three representative network-based methods, SSGC achieves the highest performances, in terms of both AUC score and the identification rates of true drug-disease pairs. The experiments with different integration strategies also demonstrate that considering several sources of data can improve the performances of the predictors. Further case studies on four diseases, the top-ranked drug-disease associations have been confirmed by KEGG, CTD database and the literature, illustrating the usefulness of SSGC.

**Conclusions:**

The proposed comprehensive similarity scores from multi-views and multiple layers and the graph-cut based algorithm can greatly improve the prediction performances of drug-disease associations.

## Background

On one hand, traditional drug development is a time-consuming and costly process with low success rate [1–3]. To speed up the process and reduce the risks and costs, drug repositioning has becoming a promising alternative for de novo drug discovery [1, 4, 5]. However, to reposition a drug might also be a haphazard process with a bit of luck, for examples, repositioning sildenafil (brand name: Viagra) from the treatment of angina to erectile dysfunction [6], repositioning minoxidil from the treatment of hypertension to hair loss [7], and so on. Thus, there are urgent needs to develop effective computational methods for drug reposition. On the other hand, the commonly used drugs for some diseases may suffer from the problems of severe side-effects or resistance, for example, the drug for Parkinson’s disease, L-dopa, has severe side effects such as dyskinesia [8]. It is necessary to find better pharmacological treatments of some diseases. Predicting drug-disease interactions is devoted to above two issues.

There are lots of methods proposed to predict the potential drug-disease relations. Some methods are based on gene expression profile data under the hypothesis that if the drug and disease have opposite expression signatures, then the drug is possible to treat that disease [9]. For instance, Sirota et al. integrated gene expression measurements from 100 diseases and 164 drug compounds, and predicted potential indications for these drugs, such as lung adenocarcinoma as the potential indications of cimetidine [10]; Jahchan et al. proposed a systematic approach to query gene expression profiles so as to identify antidepressant drugs to treat small cell cancer [11]. The vast amount of information of drugs and diseases in literature and databases make it possible to mine or infer the potential associations between drugs and diseases based on literature mining and semantic inference. Suppose that B is reported to be one of the characteristics of disease C in some literature, and drug A is reported to affect B in other literature, then it has a potential interaction between drug A and disease C [12, 13]. For example, Ahlers et al. found the potential link between the antipsychotic agents and cancer based on MEDLINE citations [14]. Since high-throughput experiments have accumulated massive data on diseases and drugs, more and more methods focus on building prediction models via machine learning strategies. For example, Gottlieb et al. proposed a logistic regression based method by integrating different information on drugs and diseases [15]; Chen et al. regarded the prediction of drug-disease associations as a recommendation problem, and adopted two recommendation algorithms to infer drug-disease interactions [16]; Liang et al. developed a Laplacian regularized sparse subspace learning (LRSSL) based method to predict drug-disease interactions by integrating drug chemical structure, drug target domain and target annotation information [17].

In recent years, the network-based prediction, which first builds a network based on the existed data and then builds the prediction model, is very promising and a few methods have been proposed, such as network-based guilt-by-association (GBA) method [4], network-based inference (NBI) method [18], random walk and network propagation based algorithm [19], and so on. Recently, Wang et al. proposed to build heterogeneous graph model HGBI for the prediction of drug-target interactions [20], and to build three-layer heterogeneous graph model (TL-HGBI) for the prediction of drug-disease interactions [21]. Even so, they did not take full advantages of the diverse information from genes, drugs, diseases, and their associations yet.

**Fig. 1:**
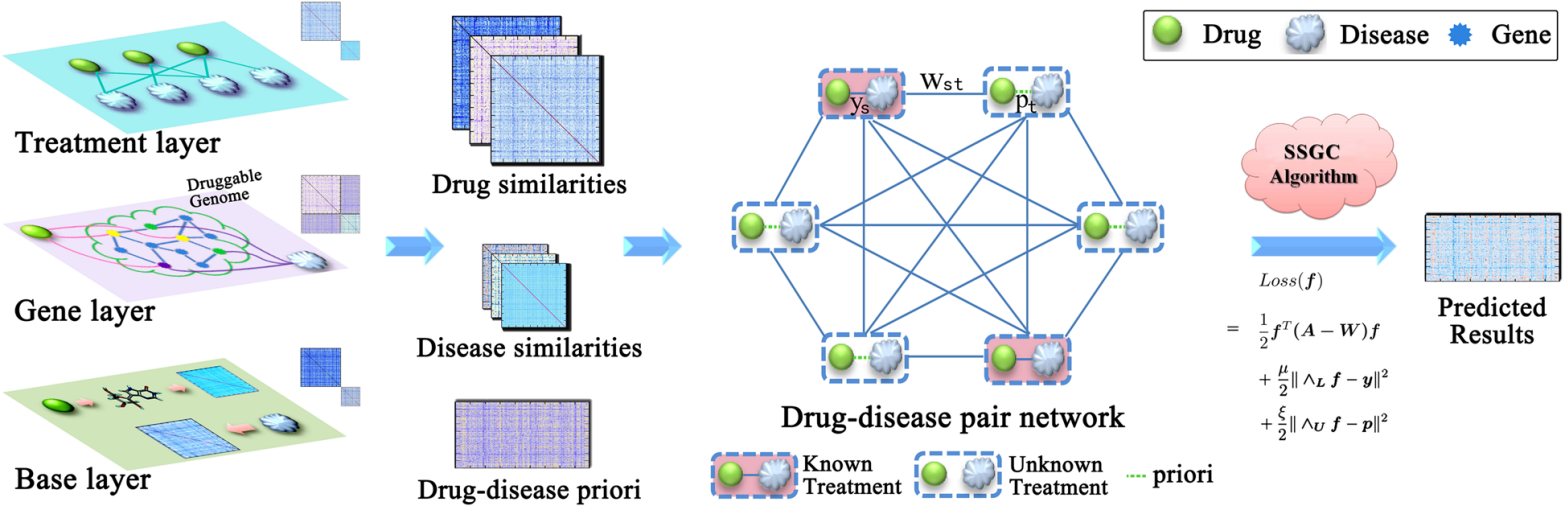
The framework of this work. Firstly, multi-sources of data, such as drug substructures, disease phenotypes, protein-protein interactions (PPI), gene profiles and network profiles, are well organized into three layers. Secondly, those information are utilized to calculate similarity scores as well as drug-disease treatment priori. Thirdly, the similarity matrices and the priori are integrated to construct drug-disease pair graph. Finally, SSGC algorithm is applied to predict drug-disease treatment relations

Since organizing heterogeneous data in a good way may contribute to the discovery of drug-disease relations [21, 22] and help to build accurate prediction models, in this work we first present a framework to integrate multiple sources/levels of data into base layer, gene layer and treatment layer. Each layer is expected to reflect one aspect of the drug-disease associations. Then we construct a novel weighted graph where a node is a drug-disease pair and an edge represents the node-node relation with the similarity score between two pairs as its weight. According to the observed data, some drug-disease pairs have known treatment relationships whereas others have not. Based on the weighted graph, we propose a semi-supervised graph cut (SSGC) algorithm to predict the drug-disease interactions that have been observed in the data yet. The overall framework is shown in Fig. 1.

## Methods

### Data collection

We have collected drugs, genes, diseases, and the interactions information from several data sources. With these data, we attempt to investigate whether there is a treatment relation within any unknown drug-disease pair.

From DrugBank (https://www.drugbank.ca) [23], we obtained the chemical structures of 1186 drugs, 1141 genes, and 4594 drug-gene associations (the polypeptides and drugs whose targets are not in human cells are not included).

From DGIdb (http://dgidb.genome.wustl.edu) [24], MINT (http://mint.bio.uniroma2.it) [25] and UniProt (http://www.uniprot.org) [26], we have collected 6988 genes, and 42162 gene-gene associations. Among the genes, 1141 genes are associated with drugs (in DrugBank), and 700 genes are associated with diseases.

From OMIM (https://omim.org) [27] and Gottlieb’s data set [15], we downloaded 449 diseases and 700 related genes that form 1365 disease-gene associations. Furthermore, 1827 treatment relations between 302 of the 449 diseases and 551 drugs [15] were also collected.

To facilitate the data integration, we organized the heterogeneous data into three layers. The base layer provides information on drug substructures and disease phenotypes; the gene layer provides genes and gene-gene associations information; and the treatment layer provides drug-disease interactions information (left part of Fig. 1).

For convenience, we suppose there are *m* drugs (*m*=1186), *n* diseases (*n*=449), *l* druggable genes (*l*=6988), and *q* drug-disease pairs (*q*=*m*×*n*) hereinafter. Moreover, we denote the *k*-order identity matrix as *I*_*k*_, matrix element multiplication and division as ⊗ and ⊘ respectively, and the shorthand for the Euclidean norm as ∥∙∥.

### Similarity calculation in the base layer

Our approach is mainly inspired by the assumption that similar drugs might treat similar diseases. Hence, similarity calculation is the key issue of our approach. Different with other methods, we first computed drug-drug and disease-disease similarities from three different aspects, corresponding to the drug structures/disease phenotypes, functional information of genes, and drug-disease treatment relationships respectively; And then we integrated three similarities into the comprehensive drug (disease) similarity.

In the base layer, we calculate the drug-drug and disease-disease similarities respectively according to drug chemical substructures and disease phenotype information.

#### Structural similarity between drugs

The SMILES (simplified molecular-input line-entry system) strings [28] for all drug structures are obtained from the DrugBank database, based on which the 2D fingerprints of the drugs are calculated via Openbabel tool [29]. Using the fingerprints information, we can calculate the Tanimoto score (the size of the intersection divided by the size of the union) [30] and use it as the structural similarity for each drug pair. Obviously, the drug-drug structural similarity matrix, denoted as *S*_*bc*_, is an *m*×*m* symmetrical matrix with diagonal elements being ones.

#### Phenotype similarity between diseases

The normalized phenotype similarity scores (ranging from 0 to 1) between diseases are obtained directly from MimMiner (http://www.cmbi.ru.nl/MimMiner/suppl.html) [31] which are constructed based on MeSH terms [32]. The *n*×*n* disease-disease phenotype similarity matrix, *S*_*bd*_, is also an symmetrical matrix with diagonal elements ones.

### Similarity calculation in the gene layer

Since diseases (drugs) associated with the same genes or genes in the same pathways are likely to have similar functional mechanism, we can measure the functional similarities of the disease (drug) pairs according to the associated genes’ information.

#### Gene-gene association measurement

Based on the gene-gene interaction network, we first measure all gene pairs distances by using all-pairs shortest path algorithm. Suppose the result gene-gene distance matrix is *D*_*g*_. For genes *i* and $\phantom {\dot {i}\!}i^{'}$, we then calculate their association according to the following Perlman’s formula [33]:

$$S_{g}\left(i,i^{'}\right) = a e^{-{bD}_{g}\left(i,i^{'}\right)} $$

where *S*_*g*_ is the *l*×*l* association matrix which is obviously symmetrical and with diagonal elements ones; *a* and *b* are two scalars that are respectively set to 0.3 and 1.0 by experience.

#### Profile similarity between drugs or diseases

We first get the profile for each drug or disease according to the drug-gene or disease-gene interaction information. The profile is represented as an *l*-dimensional vector in which every element corresponds to one gene and is encoded as either 1 or 0 indicating whether the gene associates with the drug or disease. Suppose the profiles of drugs *i* and $\phantom {\dot {i}\!}i^{'}$ are *c*_*i*_ and $\phantom {\dot {i}\!}c_{i}^{'}$, the profiles of disease *j* and $\phantom {\dot {i}\!}j^{'}$ are *d*_*j*_ and $\phantom {\dot {i}\!}d_{j}^{'}$. We then separately calculate the profile similarities according to the following two fomulas:

$$S_{gc}\left(i,i^{'}\right) = \frac{c_{i}^{T} S_{g} c_{i^{'}}} {\sqrt{c_{i}^{T} S_{g} c_{i}}\sqrt{c_{i^{'}}^{T} S_{g} c_{i^{'}}}} $$

$$S_{gd}\left(j,j^{'}\right) = \frac{d_{j}^{T} S_{g} d_{j^{'}}} {\sqrt{d_{j}^{T} S_{g} d_{j}}\sqrt{d_{j^{'}}^{T} S_{g} d_{j^{'}}}} $$

where *S*_*gc*_ is the *m*×*m* drug profile similarity matrix, and *S*_*gd*_ is the *n*×*n* disease profile similarity matrix. Obviously they are symmetrical and with elements ones on the main diagonal.

### Similarity calculation in the treatment layer

If two drugs (diseases) share some diseases (drugs), they might be similar. Therefore, the known drug-disease associations can also be utilized to calculate the drug-drug (disease-disease) similarities. According to the drug-disease associations, we first build a drug-disease bipartite graph, and then compute the drug-drug (disease-disease) distances by using the all-pairs shortest path algorithm. The distances can easily be converted into the similarity scores according to the Perlman formula [33]:

$$S_{tc}^{'}\left(i,i^{'}\right) = ae^{-b D_{tc}\left(i,i^{'}\right)}; \ \ S_{td}^{'}\left(j,j^{'}\right) = ae^{-b D_{td}\left(j,j^{'}\right)} $$

where *D*_*tc*_ is the 551 × 551 dimensional drug distance matrix; *D*_*td*_ is the 302 × 302 dimensional disease distance matrix. Accordingly, $S_{tc}^{'}$ is the 551 × 551 dimensional drug similarity matrix; $S_{td}^{'}$ is the 302 × 302 dimensional disease similarity matrix. We set the scalars *a* and *b* to 0.9 and 1 by experience, and set the self-similarity of a drug (disease) to one.

It is noticeable that we have collected 1186 drugs and 449 diseases in all, yet we can only calculated the similarities for 551 drugs and 302 diseases in the treatment layer according to the information from Gottlieb’s data set. Therefore, we adopt the same method as in [34] to project those drugs (diseases) that do not occur in Gottlieb’s data set into a unified network similarity space. By this way, we can get all drug-drug (disease-disease) similarities from $S_{tc}^{'} \left (S_{td}^{'}\right)$. We denote the final similarity matrice in treatment layer as *S*_*tc*_ (*m*×*m* dimension) and *S*_*td*_ (*n*×*n* dimension) respectively.

### Integrating similarities from three layers

Similarity measurements respectively from three layers can be integrated via various approaches. For simplification, we just adopt the linear combination strategy in this work. More sophisticated strategies will be considered in the future. Concretely, the comprehensive drug-drug (disease-disease) similarity matrix *S*_*c*_ (*S*_*d*_) are obtained as follows.

1 $$\begin{array}{@{}rcl@{}} S_{c} &=& \alpha_{c} S_{bc} + \beta_{c} S_{gc} + \gamma_{c} S_{tc} \end{array} $$

2 $$\begin{array}{@{}rcl@{}} S_{d} &=& \alpha_{d} S_{bd} + \beta_{d} S_{gd} + \gamma_{d} S_{td}  \end{array} $$

where *α*_*c*_,*β*_*c*_,*γ*_*c*_, *α*_*d*_,*β*_*d*_ and *γ*_*d*_ are combination weights satisfying that *α*_*c*_+*β*_*c*_+*γ*_*c*_=1 and *α*_*d*_+*β*_*d*_+*γ*_*d*_=1.

To determine the values of *α*_*c*_,*β*_*c*_,*γ*_*c*_, *α*_*d*_,*β*_*d*_ and *γ*_*d*_, a simple way to integrate the similarities is to assign equal weights to each layer. However this integration strategy has a weak point: the information from the layer with much smaller scores might be neglected due to the integration, and *vice verse*. A more rational way is to make each layer has equal contribution to the final results. In this work, we adopted the latter strategy to integrate the similarities from three layers.

### Novel weighted drug-disease pair graph

There are *m*∗*n* drug-disease pairs in all based on *m* drugs and *n* diseases, where some pairs have known treatment relationships according to the observed data whereas others have not. The aim of this work is to determine whether an unknown drug-disease pair has a treatment relationship or not. We propose to construct a novel weighted completed graph *G*=(*V*,*E*) for this purpose, where *V*={(*i*,*j*)|*d* *r* *u* *g* *i*∈[1,*m*],*d* *i* *s* *e* *a* *s* *e* *j*∈[1,*n*]}. $\phantom {\dot {i}\!}E = \left \{e_{st}| s \neq t, s=(i,j)\in \left [1,q\right ], t=\left (i^{'},j^{'}\right) \in \left [1,q\right ]\right \}$. In fact, *s*=(*i*−1)×*n*+*j*, $\phantom {\dot {i}\!}t = \left (i^{'}-1\right)\times n + j^{'}$. For every edge *e*_*st*_, we assign a weight to it as the similarity score between two nodes that is calculated as follows:

3 $$ W(s,t) = \left\{ \begin{array}{ll} S_{c}\left(i,i^{'}\right)S_{d}\left(j, j^{'}\right), & s \neq t \\ 0, & s = t \end{array}\right.  $$

where *W* is the *q*×*q* weight matrix that is symmetrical and with the diagonal elements zeros.

Obviously, In all *q* drug-disease pair nodes in the graph, some drug-disease pairs have known treatment relationships whereas others are unknown which need to be predicted.

Let *f*=(*f*_1_,*f*_2_,⋯,*f*_*s*_,⋯,*f*_*q*_)^*T*^, *f*_*s*_∈{0,1} indicates whether the drug-disease pair (*i*,*j*) has a treatment relationship or not. Then the problem of predicting the drug-disease treatment relationships could be addressed by determining the value of *f*. In this work, we consider this problem as a graph cut problem [35], and cluster all drug-disease pair nodes into two groups (treatment and non-treatment) by cutting the graph into several sub-graphs so that pairs within the same sub-graph belong to the same group.

### Semi-supervised graph cut approach

Suppose the treatment label matrix obtained from the data be *Y* (*m*×*n*). *Y*_*ij*_ is 1 if drug *i* can treat disease *j*, otherwise 0. If drug *i* relates to genes or pathways that also associated with disease *j*, then the drug would potentially treat the disease. We take this priori knowledge into consideration by introducing a priori matrix *P* (*m*×*n*), where the element *P*_*ij*_ is calculated as the following:

4 $$ P_{ij} = \left\{ \begin{array}{ll} \frac{c_{i}^{T} S_{g} d_{j}}{\sqrt{c_{i}^{T} S_{g} c_{i}}\sqrt{d_{j}^{T} S_{g} d_{j}}}, & Y_{ij} = 0 \\ 0, & Y_{ij} = 1 \end{array}\right.  $$

Equation (4) illustrates that we only consider the priori values of unknown drug-disease pairs.

Let ∧_*L*_(Labeled) and ∧_*U*_(Unlabeled) are two *q*×*q* diagonal matrices indicating the treatment states of drug-disease pairs observed from the data set; *p*=(*p*_1_,*p*_2_,⋯,*p*_*s*_,⋯,*p*_*q*_)^*T*^(*p*_*s*_=*P*_*ij*_); *y*=(*y*_1_,*y*_2_,⋯,*y*_*s*_,⋯,*y*_*q*_)^*T*^(*y*_*s*_=*Y*_*ij*_). Obviously, *y* is the diagonal vector of matrix ∧_*L*_; ∧_*U*_=*I*_*q*_−∧_*L*_; and $\wedge _{L}^{k} = \wedge _{L}$, $\wedge _{U}^{k} = \wedge _{U}$; ∧_*L*_ *y*=*y*, ∧_*U*_ *p*=*p*.

We define a loss function *L* *o* *s* *s*(*f*) to be minimized as follows:

5 $$ {{} \begin{aligned} Loss(f) =& \frac{1}{4}\sum_{s,t} W_{st} (f_{s} - f_{t})^{2} +\\& \frac{\mu}{2}\|\wedge_{L}f-y\|^{2} + \frac{\xi}{2}\|\wedge_{U}f-p\|^{2} \end{aligned}}  $$

Where *μ* and *ξ* are two parameters. Obviously, in order to minimize *L* *o* *s* *s*(*f*), *f* should meet the requirements that similar drug-disease pairs should have similar treatment relationships; the derived treatment relationships should be in accord with the known observed facts and also should be inclined to consistent with the priori knowledge. In this work, we set *μ*>*ξ*>0 with the consideration that violating the observed facts would receive greater penalty than out of the priori knowledge. Obviously, the *f* with the minimal *L* *o* *s* *s*(*f*) corresponds to the optimal graph cut.

Let *A* be a *q*×*q* diagonal matrix with diagonal vector *a*=(*a*_1_,*a*_2_,⋯,*a*_*s*_,⋯,*a*_*q*_), where $a_{s} = \sum _{t} W_{st} = \sum _{i^{'}} S_{c} \left (i,i^{'}\right) \sum _{j^{'}} S_{d} \left (j,j^{'}\right) -1$. Then we have

6 $$  \frac{1}{4} \sum_{s,t} W_{st} (f_{s} - f_{t})^{2} = \frac{1}{2} f^{T} (A-W)f  $$

Suppose *L*=*A*−*W*, obviously *L* is the Laplace matrix of *G*, and the normalized matrix [36] is $\overline {L} = A^{-1/2} LA^{-1/2} = I_{q} - A^{-1/2} WA^{-1/2}$. Let *S*=*A* ^−1/2^ *W* *A* ^−1/2^, then we have $\overline {L} = I_{q} - S$.

Hence, Eq. (5) turns into the following equation:

7 $$  Loss(f) = \frac{1}{2} f^{T} \overline{L} f + \frac{\mu}{2}\|\wedge_{L} f-y\|^{2} + \frac{\xi}{2}\|\wedge_{U} f-p\|^{2}  $$

According to the original definition of *f*, every element *f*_*s*_∈{0,1}, which makes the problem of minimizing *L* *o* *s* *s*(*f*) be NP-hard. We therefore relax the constraint and let *f*_*s*_∈[0,1] hereinafter. Correspondingly, we can get the derivative of *L* *o* *s* *s*(*f*):

8 $$ \nabla Loss(f) = (I_{q} + \mu \wedge_{L} + \xi \wedge_{U}) \ f - Sf - (\mu y+\xi p)  $$

To minimize *L* *o* *s* *s*(*f*), ∇*L* *o* *s* *s*(*f*) is expected to be 0. According to the gradient descent algorithm, ∇*L* *o* *s* *s*(*f*)=0 equals that Eq. (9) is convergent (*α* is a learning rate).

9 $$ \begin{aligned} f^{(k+1)} &= f^{(k)} - \alpha \nabla Loss(f)|_{f=f^{(k)}} \\ &= \alpha\left[(\mu-\xi)\wedge_{U}+S\right]f^{(k)} +(1-\alpha)\widehat{y} \end{aligned}  $$

Fortunately, Eq. (9) is convergent when setting *α*=1/(1+*μ*), $\widehat {y} = y+\frac {\xi }{\mu }p$ and $f^{(0)}=\widehat {y}$ according to [37]. It is expected to minimize *L* *o* *s* *s*(*f*) by repeating the iterative process until Eq. (9) converges. However, we find that the memory consumption is too large when running the iteration because of the extreme large matrix *S* (for example, if *n*=10^3^,*m*=10^3^, then the dimension of *S* is 10^12^).

Now that directly calculating *Sf* in Eq. (9) is space expensive, we provide a method to calculate it without explicit storage consumption. Let *F* and $\widehat {A}$ are two *n*×*m* auxiliary matrices respectively with elements as

$$\begin{aligned} F_{ij} &= f_{s} = f_{(i-1)\times n + j} \\ \widehat{A}_{i,j} &= \sqrt{a_{s}} = \sqrt{a_{(i-1) \times n +j}} \end{aligned} $$

Let $\widetilde {A} = \widehat {A}\otimes \widehat {A}$, then we have $(A^{-1}f)_{s} =(F\oslash \widetilde {A})_{ij}$ and

$$\begin{aligned} & \,\,\,\,\,\,\,\left[\left(A^{-1}+S\right)f\right]_{s}\\ &= \left[A^{-1/2}(I_{q} + W)A^{-1/2}f\right]_{s} \\ &= \sum_{t} \frac{(I_{q} + W)_{st}f_{t}}{\sqrt{a_{s}}\sqrt{a_{t}}} \\ &= \frac{1}{\widehat{A}_{ij}} \sum_{i^{'},j^{'}} \frac{S_{c}\left(i,i^{'}\right)F_{i^{'}j^{'}}S_{d}\left(j^{'}, j\right)}{\widehat{A}_{i^{'}j^{'}}} \\ &= \frac{1}{\widehat{A}_{ij}}S_{c}(i, \ast)\left(F\oslash\widehat{A}\right)S_{d}(\ast, j) \end{aligned} $$

where *S*_*c*_(*i*,∗) represents the *i*-th row of matrix *S*_*c*_ and *S*_*d*_(∗,*j*) indicates the *j*-th column of matrix *S*_*d*_. Therefore, we have

10 $$  (Sf)_{s} = \left[S_{c}\left(F\oslash\widehat{A}\right)S_{d}\oslash\widehat{A}-F\oslash\widetilde{A}\right]_{ij}  $$

Equation (10) implies that we can compute *Sf* with a space complexity *Θ*(max(*n* ^2^,*m* ^2^)), rather than *Θ*((*n* *m*)^2^), which enables the iteration process to go through on the desktops.

To sum up, the framework to find the optimal graph cut is listed in Algorithm 1.



## Results

### Redundancy check of the data set

**Fig. 2:**
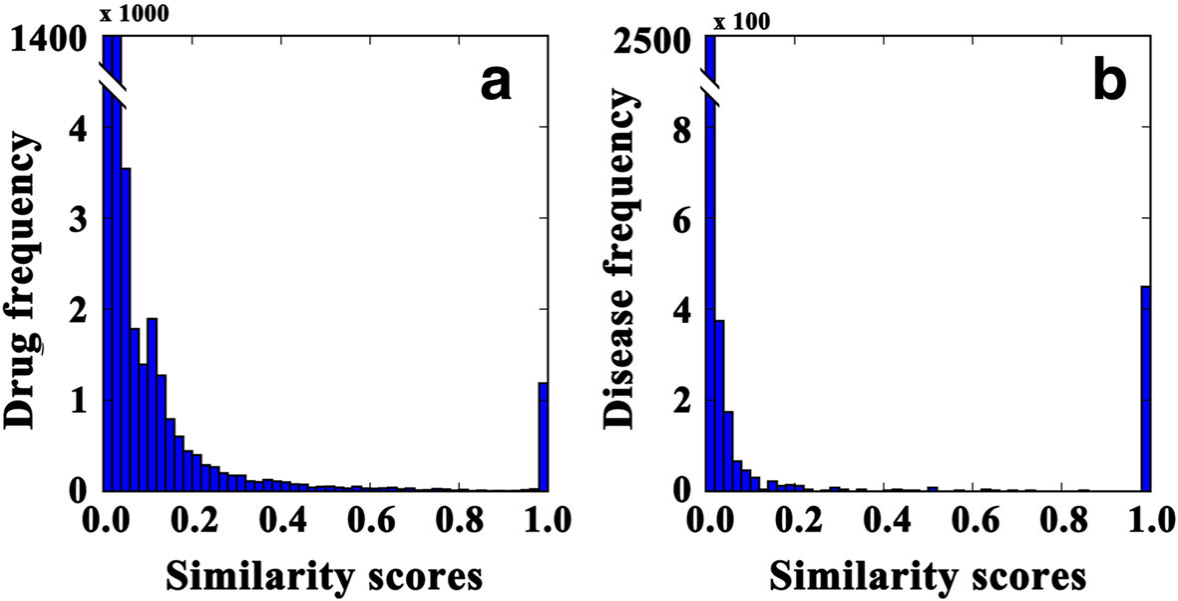
Similarity scores distribution. **a** Similarity scores distribution of drugs. **b** Similarity scores distribution of diseases. The right most bars of both (**a**) and (**b**) indicate self similarity scores

We desire to check the redundancy of the data set, since high redundant data set could lead to worse generalization. The redundancy is measured by similarity score distribution of drugs and diseases. Figure 2 a shows the similarity scores distribution of drugs. Obviously, the number of drug pairs with high similarity score is small (only 0.12% of the drug pairs have similarity scores larger than 0.5) and the majority similarity scores are around zeros. Figure 2 b demonstrates the similarity scores distribution of diseases, and the case is similar. Only 0.23% of the disease pairs have similarity scores larger than 0.5, and the majority of the scores are around zeros. Therefore, we can conclude that the majority similarity scores of both drug pairs and disease pairs are small and the redundancy of data set is negligible.

### Rationality validation by guilt-by-association assumption

As multiple sources of information has been collected and organized into three layers based on the inherent relationships, we wish to illustrate the rationality and validity of the collected information as well as the way to organize them by guilt-by-association (GBA) principle. The basic assumption of GBA is that similar drugs are inclined to be associated with similar diseases and vice versa, which implies two aspects: the drugs treating the same disease share structure/network properties and the diseases treated by the same drug also share phenotype/network properties. Therefore, similarity scores of drugs (diseases) which share some diseases (drugs) should be apparently greater than those which don’t share any diseases (drugs). Obviously, the validation results (Table 1) on the data support the GBA assumption. At the same time, the GBA ratios increase along with the layers, it is reasonable that the higher layer integrates more information.

**Table 1:** GBA analysis

	Base layer			Gene layer			Treatment layer		
	avg-same	avg-diff	ratio	avg-same	avg-diff	ratio	avg-same	avg-diff	ratio
Drug	0.25	0.17	1.47	0.29	0.12	2.41	0.33	0.06	5.50
Disease	0.23	0.10	2.30	0.40	0.13	3.08	0.32	0.05	6.40

avg-same: represent the overall average similarity scores of drugs/diseases which share some diseases/drugs. avg-diff: represent the overall average similarity scores of drugs/diseases which don’t share any diseases/drugs. ratio = avg-same / avg-diff

### Setting of thresholds and combinations weights

Previous studies imply that small similarity scores are usually noise data which provide little information and sometimes even have adverse effect to the prediction performance [20, 21]. Therefore, we chose thresholds to cut off the small similarity scores. However, taking the thresholds together, there are 12 parameters in Eqs. (1) and (2) in all, which makes it impractical to search all the parameter space to get the optimal parameter settings. For feasibility, we set the parameters based on two principles: (1) each layer has close GBA ratio; and (2) each layer has nearly equal contribution to the ultimate similarity matrices.

#### Thresholds setting based on GBA assumption

We want to let each layer have similar GBA ratio. Since the treatment layer achieves the highest GBA ratios (Table 1), we set the similarities thresholds for *S*_*tc*_,*S*_*td*_ to zeros and then accordingly choose the thresholds for other two layers so that three layers have similar GBA ratios. As a result, the thresholds of *S*_*bc*_,*S*_*gc*_,*S*_*bd*_ and *S*_*gd*_ are set to 0.1, 0.01, 0.14 and 0.01 respectively.

#### Integrating weights setting based on equal contribution strategy

We want to let each layer have nearly equal contribution to the ultimate similarity matrices. After choosing of thresholds, the average of each matrix (*S*_*bc*_,*S*_*gc*_,*S*_*tc*_,*S*_*bd*_,*S*_*gd*_ and *S*_*td*_) are calculated to be 0.017, 0.028, 0.057, 0.006, 0.028 and 0.038 respectively. Accordingly we can obtain the combination weights by setting equal contributions to each layer. If the average of one layer is small, we assign a large weight to enhance its final effect, on the same time, if the average of one layer is large, we assign a small weight to weaken its final effect. By this strategy, we set *α*_*c*_,*β*_*c*_ and *γ*_*c*_ to 0.53, 0.32 and 0.15; and *α*_*d*_,*β*_*d*_ and *γ*_*d*_ to 0.72, 0.16 and 0.12 respectively.

### Evaluating the performance of SSGC

Since SSGC is a network-based approach, we compared it with three network-based methods (NBI, HGBI and TL-HGBI) on Gottlieb’s data set using 10-folds cross validation [15]. For fairness, we optimize the parameters for each method by grid search: *μ*=4 and *ξ*=0.67 for SSGC, *α*=0.7 for HGBI and *α*=0.2 for TL-HGBI.

Using each of four algorithms, we can respectively predict a candidate drug list for every disease. We consider each observed drug-disease pair in the data set has true treatment relation (positive sample). Since we only have positive samples, the calculation of the receiver operating characteristic (ROC) curve is different from the standard approach [21]. For an observed drug-disease pair in the data set, if the treatment relation value (obtained from *F*) is greater than the threshold, then it is regarded as a true positive (TP), otherwise a false negative (FN). For other pairs not observed in the data set, if the value is above the threshold, then it is regarded as a false positive (FP), othervise a true negative (TN). In this experiment, the threshold is set 0.05. Accordingly, we can calculate the true positive rate (TPR) and false positive rate (FPR) for a given threshold as follows:

$$TPR = \frac{TP}{TP+FN};\;\;\;\;FPR = \frac{FP}{FP+TN} $$

**Fig. 3:**
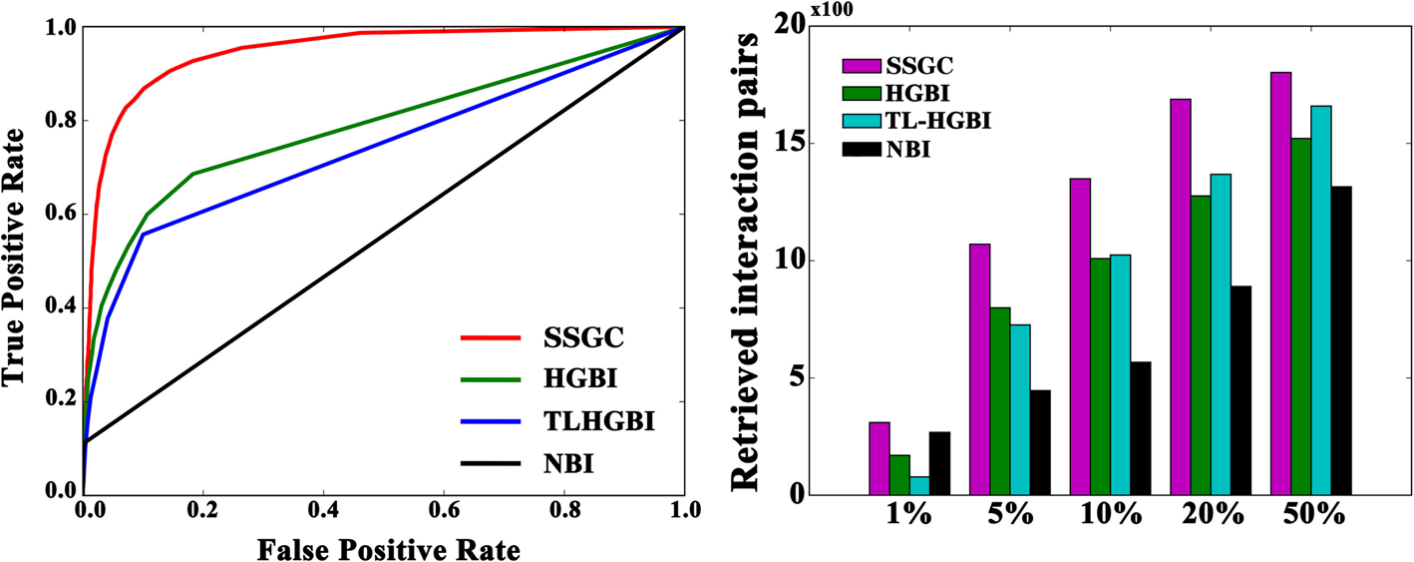
Performance evaluation. The left panel is the ROC curves of original NBI, HGBI, TL-HGBI and SSGC. The right panel is the numbers of correctly retrieved disease-drug interactions with respect to different percentiles

As shown in Fig. 3 (left), SSGC method obtains higher AUC score than the compared approaches.

At the same time, we investigate the number of correctly retrieved known drug-disease pairs among the top ranked prediction results. Figure 3 (right) shows that SSGC performs the best. For example, among the 1827 known drug-disease associations, 310 of them are retrieved among the top 1% ranked predictions by SSGC, whereas only 170 (78) of them are retrieved by HGBI (TL-HGBI).

### Investigating the integration strategy

In order to investigate whether our comprehensive similarities combination strategy contributes to the good performance of SSGC, we try to modify the compared methods so that they can adopt the same strategies. As HGBI and TL-HGBI also utilize drug-drug and disease-disease similarities to infer drug-disease interactions, it is easy to modify them to employ the combined comprehensive similarities as our method does. At the same time, SSGC can be turned to partly or fully adopt the comprehensive similarities. After the modification, we can investigate three methods in the way that multiple layers of data are added gradually. Because NBI method only makes use of the topology structure of drug-disease association network, we do not include it in this comparing experiment.

The experiment results (Table 2) show that three methods are neck and neck when just using the base layer. While along with the addition of more layers of data, SSGC and HGBI achieve considerable improvements in performance, TL-HGBI differs little at first, but its performance is also improved with information in all layers and priori added in. The results reflect the effectiveness of the comprehensive similarities obtained by our integration strategy. It is interesting to find that SSGC can be modified to be HGBI when setting $\phantom {\dot {i}\!}W_{st} = S_{c}(i,i^{'})S_{d}(j, j^{'})$, *p*=0 and *μ*=*ξ*, HGBI is a particular case of SSGC. Compared with HGBI, SSGC has better performance, which illustrates that SSGC benefits from introducing prior knowledge and removing the self-loops in the heterogeneous network.

**Table 2:** AUC scores of different algorithms modified to integrate different layers

	SSGC	HGBI	TL-HGBI
base	0.80	**0.78**	**0.74**
base + gene	0.87	0.85	0.74
base + gene + network	0.93	0.91	0.75
base + gene + network + priori	**0.95**	0.93	0.84

The values in bold are the original AUC scores of three algorithms before modification. To investigate the effect of integration strategy of SSGC, we modified three algorithms to integrate different layers and got other AUC scores listed in the table

### Validating the predicted drug-disease associations

#### Distribution of predicted values

**Fig. 4:**
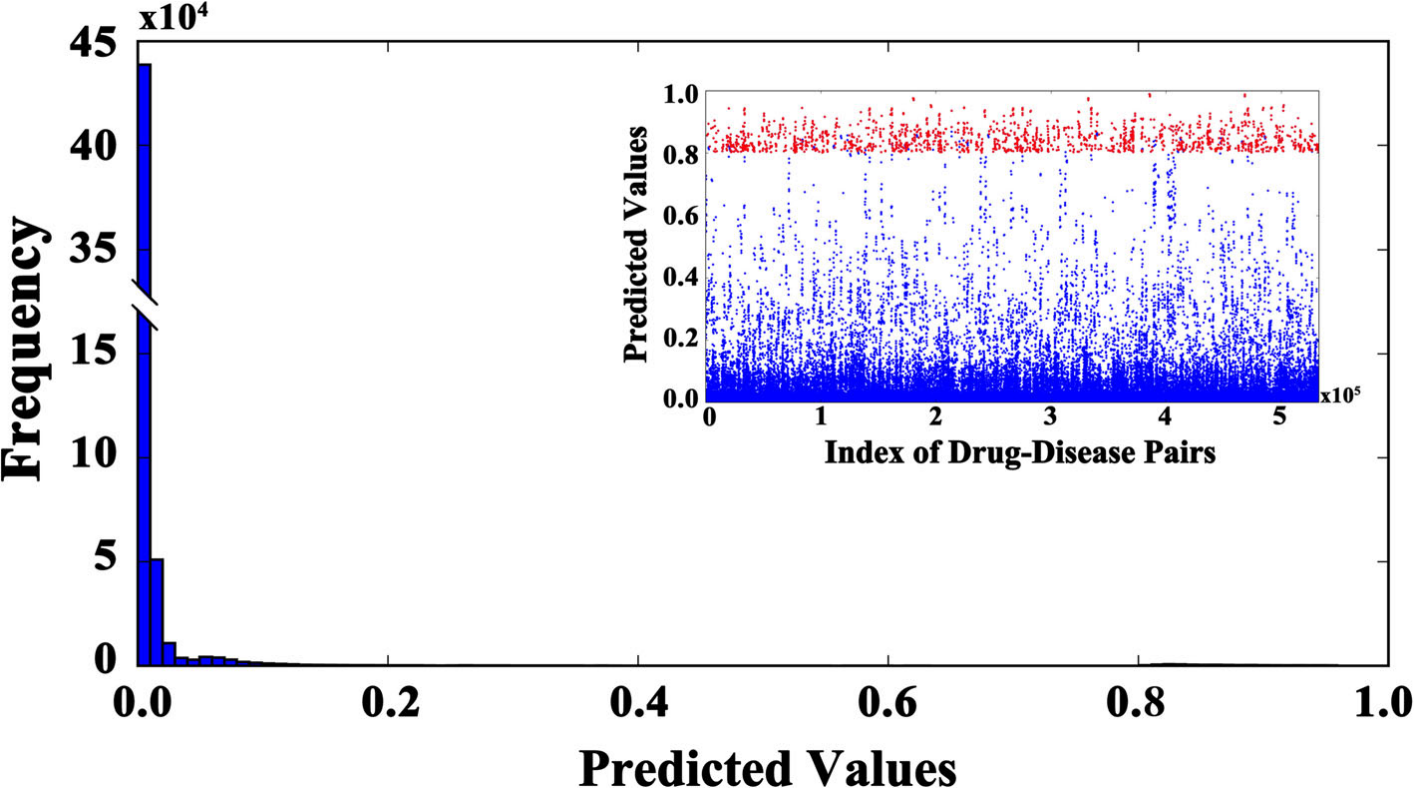
The overview of the predicted scores. The histogram represents the distribution of predicted values of all drug-disease pairs. The red and blue points in the subplot represent the predicted values of observed true treatment relations and other drug-disease pairs (unknown treatment relations) respectively

The overview of predicted interaction values is shown in Fig. 4. From the histogram we can see that the predicted values of most of drug-disease pairs are around zeros (In fact, there are only 20% of the pairs with predicted values bigger than 0.1), suggesting that only a small part of the unknown drug-disease pairs have repositioning relations, which is consistent with the common sense that the drug-disease treatments are specific. And the predicted values of drug-disease pairs with known treatment relationships are above 0.8, but it is not easy to find them in the histogram. To display the distribution of significant predicted values more clearly, we further ploted a subplot in Fig. 4. The predicted values of pairs with known treatment relations (red points) are larger than most of pairs with unknown relations (blue points), which also indicates that our method can capture the known knowledge very well.

#### Validation in tissue-specific expression data

If a disease is manifested in a tissue in which the targets (genes) of a drug are also expressed, then the drug is more likely to have treatment association with the disease. Based on this hypothesis, we utilize tissue-specific expression data to check whether our predicted results are reasonable or not. On one hand, we gather the disease-tissue associations from literature [38]. On the other hand, we get target-tissue (gene-tissue) associations from tissue-specific gene expression data [39], then further obtain the drug-tissue associations. We observe the predicted association scores of drug-disease pairs associated with the same tissue (Table 3). As expected, those scores (from 0.09 to 0.33) are far greater than the average (0.014) of all drug-disease association scores, which further shows the efficiency and rationality of SSGC to discover the potential drug-disease associations.

**Table 3:** The drug-disease pairs related to the same tissue

Tissue	Drug	Disease	Value
Pancreas	Acetylsalicylic acid (DB00945)	Diabetes Mellitus, Noninsulin-Dependent (125853)	0.20
Pancreas	Acetylsalicylic acid (DB00945)	Cystic fibrosis by Pseudomonas aeruginosa (219700)	0.32
Pancreas	Acetaminophen (DB00316)	Diabetes Mellitus, Noninsulin-Dependent (125853)	0.13
Pancreas	Acetaminophen (DB00316)	Cystic fibrosis by Pseudomonas aeruginosa (219700)	0.26
Skeletal Muscle	Acetaminophen (DB00316)	Myasthenic syndrome (601462)	0.22
Skin	Lorazepam (DB00186)	Immunodysregulation, Polyendo-crinopathy, And X-Linked Enteropathy (304790)	0.17
Testis	Lorazepam (DB00186)	Persistent Mullerian duct syndrome, type II (261550)	0.09
Testis	Alprazolam (DB00404)	Persistent Mullerian duct syndrome, type II (261550)	0.10
Testis	Acetaminophen (DB00316)	Persistent Mullerian duct syndrome, type II (261550)	0.24
Heart	Acetylsalicylic acid (DB00945)	Thrombosis, Susceptibility to thrombin defect; thph1 (188050)	0.20
Heart	Acetaminophen (DB00316)	Thrombosis, Susceptibility to thrombin defect; thph1 (188050)	0.33
Heart	Acetaminophen (DB00316)	Afibrinogenemia, congenital (202400)	0.25
Heart	Acetylsalicylic acid (DB00945)	Afibrinogenemia, congenital (202400)	0.24

### Case studies for potential drug-disease relations

We select four diseases as case studies: Huntington disease (HD, OMIM 143100), Non-small-cell lung cancer (NSCLC, OMIM 211980), Alcohol dependence (AD, OMIM 103780) and Small-cell lung cancer (SCLC, OMIM 182280). After excluding the known approved drugs which are also predicted in the results (value > 0.8), we observe other predicted top-20 ranked drugs. The investigation of the predicted drug-disease associations included three parts as follows.

#### Investigation of the pathways overlapping between the disease and drugs

For a specific disease, if the related pathways of the drugs are overlapped with those of the disease, the prediction results should be convincible. Therefore, we first extracted the disease related genes from OMIM, and the target genes of the top-20 drugs from DrugBank; and then we got the enriched pathways of the two gene sets respectively with DAVID [40, 41], and investigated the overlap between them.

**Fig. 5:**
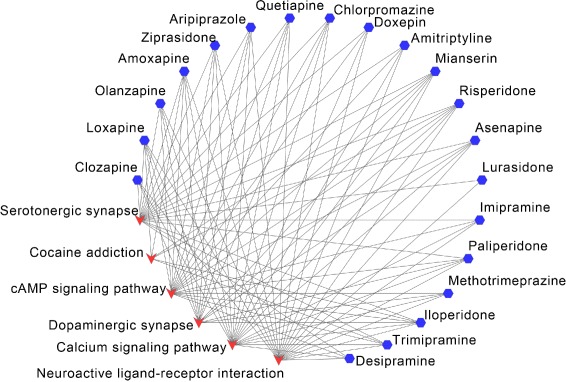
Overlapped KEGG pathways between Huntington disease and the predicted drugs. The blue hexagon nodes represent drugs predicted to treat Huntington disease, the red vee nodes represent overlapped KEGG pathways between drugs and Huntington disease

For HD, each of the top-20 ranked drugs has KEGG pathways overlapping with the disease pathways, shown in Fig. 5. The overlapped pathways are “Neuroactive ligand-receptor interaction”, “Calcium signaling pathway”, “Serotonergic synapse”, “Dopaminergic synapse”, “cAMP signaling pathway” and “Cocaine addiction”. Each drug has 5 overlapped pathways in average.

**Fig. 6:**
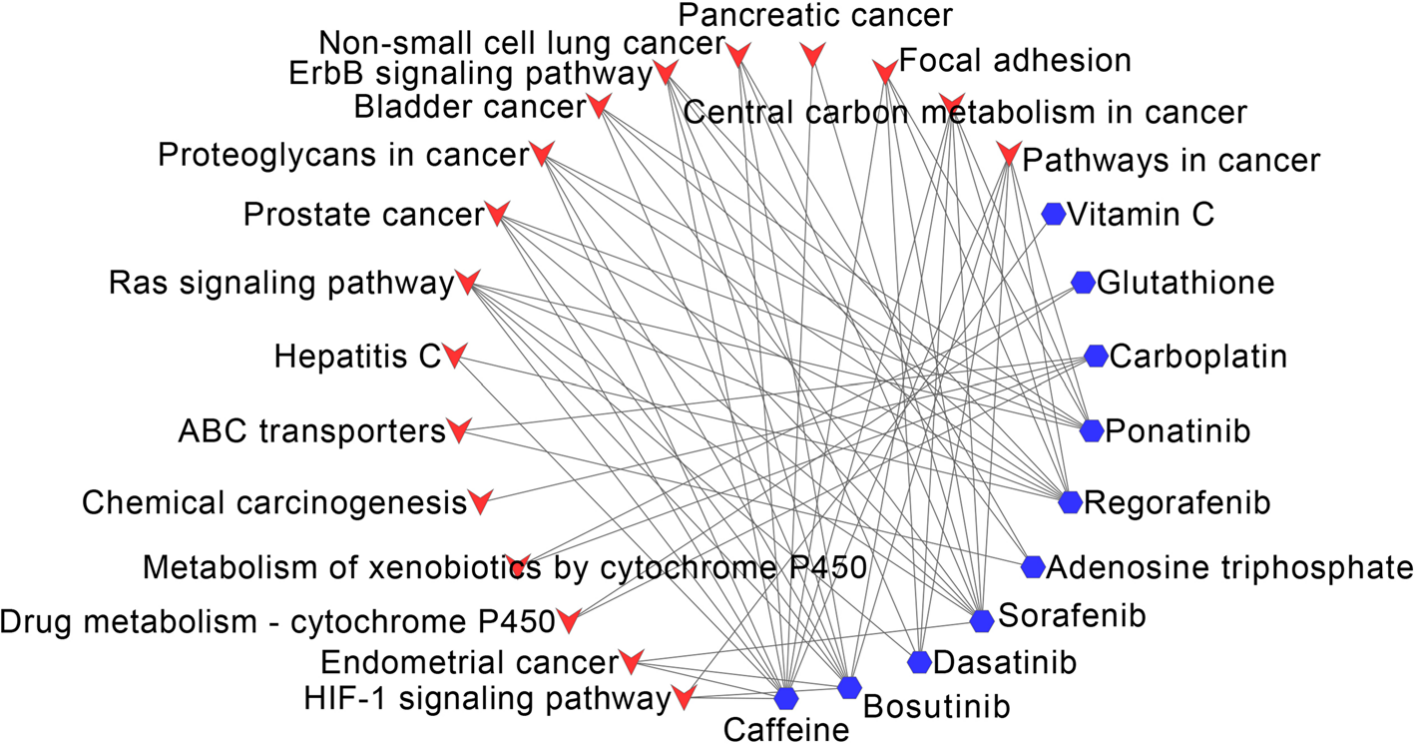
Overlapped KEGG pathways between Non-small-cell lung cancer and the predicted drugs. The blue hexagon nodes represent drugs predicted to treat Non-small-cell lung cancer, the red vee nodes represent overlapped KEGG pathways between drugs and Non-small-cell lung cancer

For NSCLC, 11 of the top-20 drugs have overlapped KEGG pathways with the disease pathways, shown in Fig. 6. Especially, Caffeine (DB00201) has 12 overlapped pathways, Sorafenib (DB00398) and Bosutinib (DB06616) have 10 overlapped pathways, Regorafenib (DB08896) has 9 overlapped pathways.

**Fig. 7:**
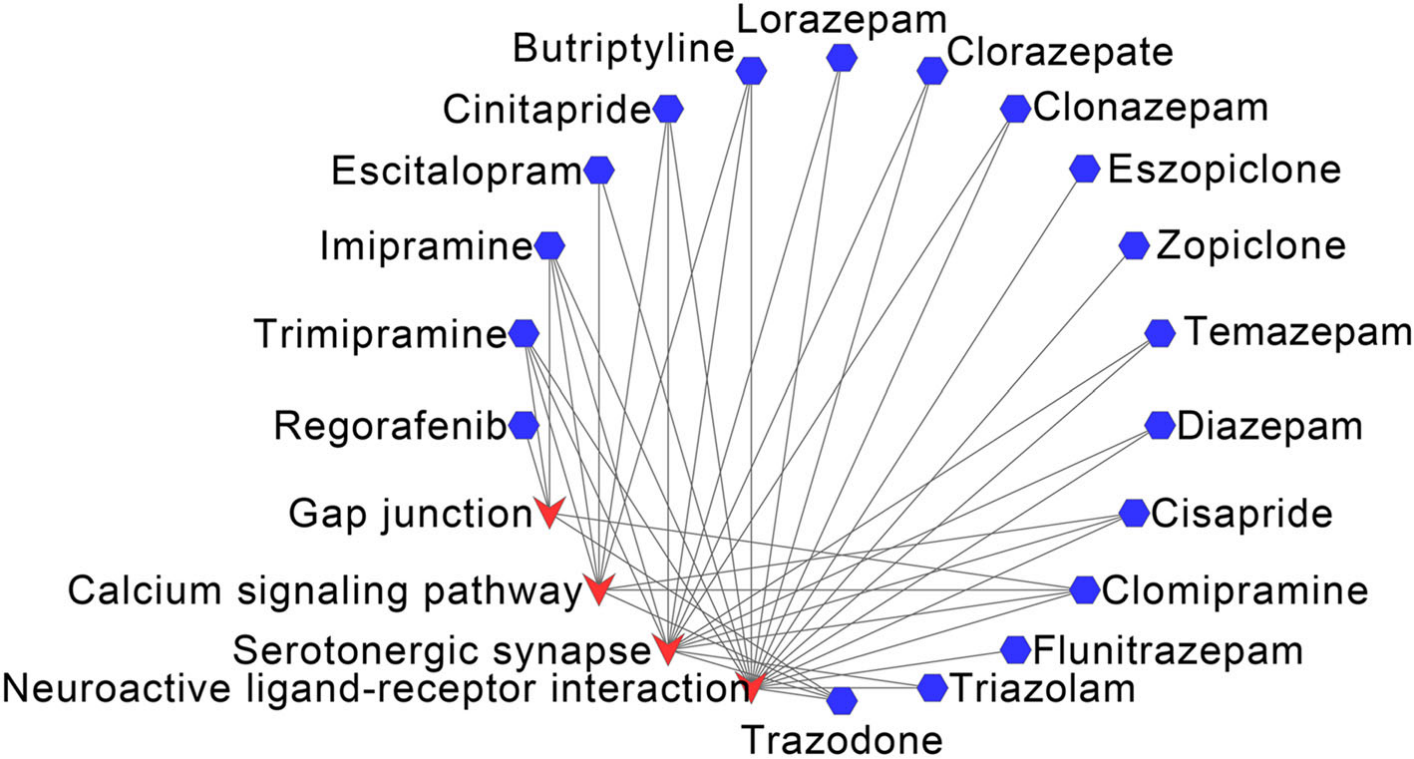
Overlapped KEGG pathways between Alcohol dependence and the predicted drugs. The blue hexagon nodes represent drugs predicted to treat Alcohol dependence, the red vee nodes represent overlapped KEGG pathways between drugs and Alcohol dependence

For AD, 18 of the top-20 drugs have overlapped KEGG pathways with the disease pathways, shown in Fig. 7. The overlapped pathways are “Calcium signaling pathway”, “Neuroactive ligand-receptor interaction”, “Serotonergic synapse” and “Gap junction”.

**Fig. 8:**
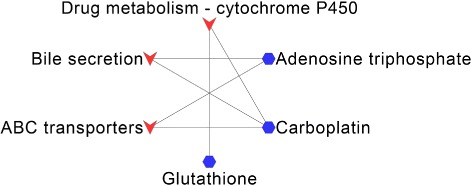
Overlapped KEGG pathways between Small-cell lung cancer and the predicted drugs. The blue hexagon nodes represent drugs predicted to treat Small-cell lung cancer, the red vee nodes represent overlapped KEGG pathways between drugs and Small-cell lung cancer

For SCLC, Carboplatin (DB00958), Adenosine triphosphate (DB00171) and Glutathione (DB00143) have overlapped KEGG pathways with the disease pathways. The overlapped pathways are “ABC transporters”, “Bile secretion” and “Drug metabolism - cytochrome P450”, which are shown in Fig. 8. Besides, Sorafenib (DB00398), Regorafenib (DB08896) and Ponatinib (DB08901) have cancer related pathways, such as “Pathways in cancer”, “Central carbon metabolism in cancer” and “Proteoglycans in cancer”.

#### Verification in CTD database

The Comparative Toxicogenomics Database (CTD, http://ctdbase.org) provides information about associations among chemicals, genes and diseases [42]. We search these four diseases in the CTD database, and their related chemicals will be listed out. These listed chemicals are associated with the disease or its descendants. If a chemical has a curated association to the disease, it will be signed with “marker/mechanism” or “therapeutic” in the “Direct Evidence” item, otherwise if the chemical just has inferred association via a curated gene interaction, there is no sign in “Direct Evidence” item. To evaluate our approach, we check the top-20 ranked drugs predicted in our method one by one to verify whether the drug-disease interaction can be found in CTD database (Table 4).

**Table 4:** The top-ranked predictions for selected diseases(Verification in CTD database)

Disease	Known drugs	Part of top-ranked predictions	Direct evidence
HD (143100)	Baclofen (DB00181)	Clozapine (DB00363, rank:01)	
	Tetrabenazine (DB04844)	Olanzapine (DB00334, rank:03)	T
		Aripiprazole (DB01238, rank:06)	T
		Amitriptyline (DB00321, rank:10)	
		Risperidone (DB00734, rank:12)	
NSCLC (211980)	Doxorubicin (DB00997)	Carboplatin (DB00958, rank:01)	T
		Adenosine triphosphate (DB00171, rank:02)	
		Glutathione (DB00143, rank:05)	
		Ponatinib (DB08901, rank:09)	
		Sorafenib (DB00398, rank:10)	
		Dasatinib (DB01254, rank:14)	
		Daunorubicin (DB00694, rank:15)	
		Epirubicin (DB00445, rank:16)	T
		Bosutinib (DB06616, rank:18)	
		Caffeine (DB00201, rank:19)	
		Cisplatin (DB00515, rank:20)	T
AD (103780)	Citalopram (DB00215)	Lorazepam (DB00186, rank:04)	T
	Chlordiazepoxide (DB00475)	Diazepam (DB00829, rank:10)	
	Acamprosate (DB00659)	Clomipramine (DB01242, rank:13)	
	Naltrexone (DB00704)	Flunitrazepam (DB01544, rank:14)	
	Disulfiram (DB00822)	Adenosine triphosphate (DB00171, rank:17)	
	Ondansetron (DB00904)	Trazodone (DB00656, rank:18)	
		Imipramine (DB00458, rank:20)	
SCLC (182280)	Cisplatin (DB00515)	Carboplatin (DB00958, rank:01)	T
	Methotrexate (DB00563)	Adenosine triphosphate (DB00171, rank:02)	
	Teniposide (DB00444)	Irinotecan (DB00762, rank:04)	T
	Etoposide (DB00773)	Glutathione (DB00143, rank:07)	
	Topotecan (DB01030)	Doxorubicin (DB00997, rank:09)	T
		Daunorubicin (DB00694, rank:11)	
		Sorafenib (DB00398, rank:13)	
		Ponatinib (DB08901, rank:16)	
		Epirubicin (DB00445, rank:18)	T

In the “Direct Evidence” item, according to the instructions in CTD database, “T” means “therapeutic”, i.e., the drug has a curated association to the disease, other top-ranked drugs aren’t signed with “T” in this table means that they have an inferred association via a curated gene interaction

As shown in Table 4, Five drugs are associated with HD, Olanzapine (DB00334) and Aripiprazole (DB01238) have curated association to HD, which are signed with “T” in the “Direct Evidence” item. Eleven drugs are associated with NSCLC, Carboplatin (DB00958), Epirubicin (DB00445) and Cisplatin (DB00515) have curated association to NSCLC. Seven drugs have association to AD, Lorazepam (DB00186) has curated association to AD. Nine drugs are associated with SCLC, Carboplatin (DB00958), Irinotecan (DB00762), Doxorubicin (DB00997) and Epirubicin (DB00445) have curated association to SCLC.

#### Verification in literature

To further examine the predicted results, we check them using literature support, and list out the drugs which have been verified in the published papers (Table 5). Among the top ranked drugs, six drugs have been reported in the treatment of HD [43–48]; three drugs have been found to treat NSCLC [49–51]; the study of Butriptyline (DB09016) on AD has already been reported by Pani *etc* [52], and the clinical trial of drug Lorazepam (DB00186) on AD has already been done [53]; Carboplatin (DB00958), Irinotecan (DB00762), Doxorubicin (DB00997) and Epirubicin (DB00445) have already been studied to treat SCLC [54–58].

**Table 5:** The top-ranked predictions for selected diseases(Verification in literature)

Disease	Known drugs (DrugBank IDs)	Part of top-ranked predictions
HD (143100)	Baclofen (DB00181)	Clozapine (DB00363, rank:01)
	Tetrabenazine (DB04844)	Olanzapine (DB00334, rank:03)
		Ziprasidone (DB00246, rank:05)
		Aripiprazole (DB01238, rank:06)
		Quetiapine (DB01224, rank:07)
		Risperidone (DB00734, rank:12)
NSCLC (211980)	Doxorubicin (DB00997)	Carboplatin (DB00958, rank:01)
		Epirubicin (DB00445, rank:16)
		Cisplatin (DB00515, rank:20)
AD (103780)	Citalopram (DB00215)	Butriptyline (DB09016, rank:03)
	Chlordiazepoxide (DB00475)	Lorazepam (DB00186, rank:04)
	Acamprosate (DB00659)	
	Naltrexone (DB00704)	
	Disulfiram (DB00822)	
	Ondansetron (DB00904)	
SCLC (182280)	Cisplatin (DB00515)	Carboplatin (DB00958, rank:01)
	Methotrexate (DB00563)	Irinotecan (DB00762, rank:04)
	Teniposide (DB00444)	Doxorubicin (DB00997, rank:09)
	Etoposide (DB00773)	Epirubicin (DB00445, rank:18)
	Topotecan (DB01030)	

All above results have demonstrated the effectiveness of our approach to discover the potential drug-disease interactions.

## Discussion and conclusion

In this paper, we propose a novel method, SSGC, to uncover the potential associations between drugs and diseases. The main contributions are as follows: Firstly, we have presented a hierarchial framework to integrate multiple source of data, including information of drug substructures, disease phenotypes, gene-gene interactions, and known drug-disease treatment relationships. The integration framework can be easily extended to integrate more data. Secondly, we measured the comprehensive similarities of drugs and diseases from multi-view and multiple layers, which is different with many other methods that just obtain the similarity from the chemical structure and the disease phenotype. The base layer reflects the drug structural similarity and disease phenotype similarity, which are the original features. The gene layer reflects the functional similarities of drugs and diseases, which are calculated based on the assumption that diseases (drugs) associated with some common genes or gene pathways might have analogous functional mechanism. The treatment layer takes the known drug-disease relationships into account, which can improve the similarities of drugs and diseases. Therefore, the comprehensive similarities can improve the prediction accuracy and are easily interpretable. Thirdly, we model the prediction as a graph cut problem, and develop a semi-supervised algorithm, SSGC, to resolve it. The experimental results imply that SSGC significantly outperforms three representative approaches. Besides, KEGG pathway enrichment analysis and the validations via CTD database and literature also demonstrated that SSGC is useful to predict the potential associations between drugs and diseases. In fact, the proposed SSGC algorithm can also be used in other recommendation systems, such as recommending products to customers.

Of course, there is a long way to go in the process of drug discovery. And there are many other types of data (side effect data of chemicals, clinical symptoms and signs, and so on) could be utilized to predict drug-disease interactions. For example, Rastegar-Mojarad et al. utilized phenome-wide association studies (PheWAS) data and further expanded the horizon for the prediction of drug-disease interactions [59]. However, how to fuse multiple sources of data more properly and rationally and how to develop prediction models with better performance and interpretability are still full of challenges.
